# Malignant nerve sheath tumour of omentum and the “omental vascular pedicle sign”

**DOI:** 10.1259/bjrcr.20150037

**Published:** 2016-01-15

**Authors:** Udit Chauhan, Alok Kumar Udiya, Shailesh M Prabhu, Gurucharan S Shetty

**Affiliations:** Department of Radiodiagnosis, Lady Hardinge Medical College, New Delhi, India

## Abstract

Primary malignant peripheral nerve sheath tumour (MPNST) is an uncommon malignant tumour that arises from the peripheral nerves. Most of these tumours arise in the regions of the trunk, head and neck, or extremities and are rarely seen in the abdomen. In this report, we describe a case of MPNST of the greater omentum, which, to the best of our knowledge, is only the second case reported in the literature. MPNST is an uncommon tumour that can show local invasion and has a high risk of recurrence. We also discuss the utility of “omental vascular pedicle sign” to help establish the omental origin of intra-abdominal masses.

## Summary

The greater omentum is a mobile peritoneal drape in relation to the greater curvature of the stomach and is sometimes referred to as the policeman of the abdomen. Numerous primary and secondary tumours can arise from the greater omentum. Among them, malignant peripheral nerve sheath tumour (MPNST) is an uncommon entity. Identifying the site of origin of an intra-abdominal mass is critical in further management and the “omental vascular pedicle sign” helps in determining the omental origin of an intra-abdominal mass.

## Case report

**Figure 1.**
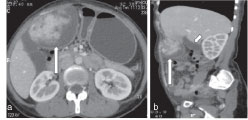
Axial (a) and coronal (b) contrast-enhanced CT images of the abdomen reveal a large solid cystic mass lesion with an intensely enhancing solid component seen in the right upper abdomen in the subhepatic location (long arrows in a and b). The lesion is seen separately from the gallbladder (short arrow in b). The lesion also shows evidence of loss of fat planes with the adjacent anterior abdominal wall suggesting invasion.

**Figure 2.**
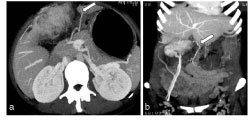
Axial (a) and coronal (b) maximum intensity projection contrast-enhanced CT image of the abdomen shows a dilated omental vein (arrows) draining the mass lesion into the superior mesenteric vein, thereby depicting the “omental vascular pedicle sign” and suggesting an omental origin of the mass lesion. The mass was excised and histopathology revealed it to be a malignant peripheral nerve sheath tumour of the greater omentum.

A 20-year-old female presented to the emergency department with a 5-day history of fever, pain and lump in the right hypochondrium. The laboratory examination was unremarkable except for mild leukocytosis. This was followed by an ultrasound examination that revealed a well-defined, round-to-oval, heteroechoic, *part solid and part cystic* lesion with internal vascularity in the subhepatic region, with loss of planes with the anterior abdominal wall muscles. The mass was seen separately from the gallbladder, which showed multiple intraluminal calculi with normal wall thickness. No other significant finding was seen in the abdomen. This was followed by a contrast-enhanced CT scan of the abdomen that revealed a large (approximately 7 × 6 cm sized) solid cystic mass lesion in the right subhepatic region showing intensely enhancing solid areas with peripheral cystic non-enhancing areas (Figure 1a,b). The mass showed loss of fat planes, with focal infiltration of the adjacent anterior abdominal wall muscles. Arterial supply to the mass was from a branch of the right gastroepiploic artery, while venous drainage was *via* the superior mesenteric vein through the right gastroepiploic vein (Figure 2). Owing to its drainage into the omental veins (“omental vascular pedicle sign”), the origin of the mass was ascertained to be from the greater omentum. Loss of fat planes with the anterior abdominal wall muscles suggested a possible malignant aetiology. Based on the imaging findings, a primary diagnosis of malignant omental mass was suggested. The patient was operated on and underwent wide local excision. *Intraoperative appearance* confirmed the omental origin of the mass with other findings being similar to those suggested by the CT scan. Gross pathological examination showed a fleshy mass with white tan surface and areas of haemorrhage measuring approximately 7 × 6 cm. Histopathological examination of the mass showed the typical appearance of alternating hypercellular and hypocellular areas, with cells arranged in a fascicular pattern within the hypercellular areas. Immunohistochemistry of the tumour cells showed positive staining for S100 and negative results for α-smooth muscle actin, desmin, c-kit, and cluster of differentiation 34, which was suggestive of a MPNST. The margins of the resected specimen were negative on microscopic examination. As the patient belonged to the reproductive age group, she was kept under regular follow-up. and after a short period of 6 months, she developed local recurrence in the abdominal wall. The patient subsequently underwent chemoradiotherapy and is on follow-up.

## Discussion

The greater omentum is a double layer of peritoneum that hangs from the greater curvature of the stomach and the proximal part of the duodenum and extends up to the level of the symphysis pubis, covering the small bowel. The greater omentum is composed mainly of fatty tissue between the peritoneal layers with traversing arteries, nerves, veins and lymphatics.^1,2^

The arterial supply of the omentum originates from the right and left gastroepiploic arteries that arise from the gastroduodenal and splenic arteries, respectively.^1^ The venous drainage runs parallel to the arteries and empties into the portal system. The right gastroepiploic vein drains into the superior mesenteric vein and the left gastroepiploic vein into the splenic vein.^3^

**Figure 3.**
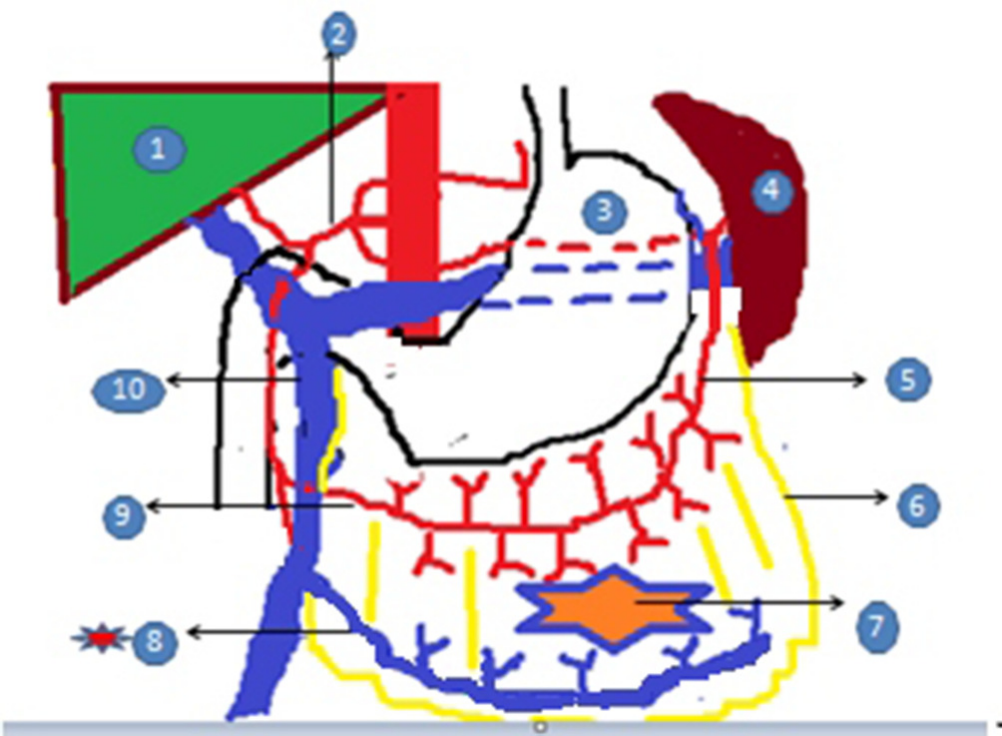
Schematic diagram of the “omental vascular pedicle sign”. 1, liver; 2, coeliac trunk; 3, stomach; 4, spleen; 5, left gastroepiploic artery; 6, greater omentum; 7, omental mass; 8, right gastroepiploic vein (red star) omental vascular pedicle; 9, right gastroepiploic artery; 10, superior mesenteric vein.

With the advent of multidetector CT scanners, it is now possible to better delineate the omental arterial blood supply and venous drainage. Karcaaltincaba et al^3^ first described the “omental vascular pedicle sign” to suggest an omental origin of an intra-abdominal mass lesion and defined it as the presence of a dilated omental vein associated with a mass located in the omentum. This sign (Figure 3) is useful for localizing the origin of a mass with invasive trans-spatial extensions, particularly in patients with less intra-abdominal fat, as was seen in our case. The caveat lies in the fact that omental vascular pedicle sign can be seen with both primary and secondary omental lesions and therefore merely suggests a possible omental origin.^3^

Omental tumours can either be primary or secondary in nature, with secondary tumours being more common. Primary omental tumours are uncommon and include various types of tumours such as gastrointestinal stromal tumours, leiomyosarcomas, leiomyomas, fibrosarcomas, liposarcomas, desmoid tumours, nerve sheath tumours, mesotheliomas, fibromas and myosarcomas.^2,3^ Among secondary tumours, the common ones are carcinomatosis, pseudomyxoma peritonei and lymphomatosis. Various tumour-like processes can also involve the greater omentum, which include tuberculous peritonitis, disseminated histoplasmosis, inflammatory pseudotumour, endometriosis, gliomatosis peritonei and osseous metaplasia.^2^

Diffuse and generalized disease processes, which could be primary, secondary or tumour-like, do not pose much problem with respect to their localization. The dilemma arises in cases of solitary masses such as nerve sheath tumours (benign and malignant), metastases, leiomyomas, leiomyosarcomas, etc. with respect to their localization to the omentum or the mesentery, especially when it shows invasion of the surrounding structures. Localization of the tumour to one of these sites not only helps in narrowing down the radiological differential diagnosis but also in proper preoperative surgical planning, which is of paramount importance in resection of potentially resectable masses.

MPNSTs are uncommon soft tissue tumours that arise from the peripheral nerves. Most of these tumours arise in the regions of the trunk, head and neck, or extremities and are very rarely seen in the abdomen.^4^ There have been isolated reports of primary and secondary MPNST arising from the omentum.^4–6^ Miguchi et al^4^ reported the first case of a primary MPNST arising from the greater omentum. They described it in a 71-year-old male patient who was otherwise asymptomatic except for pain in the lower abdomen. His CT scan showed a large, well-defined, lobulated mass with central low density areas and peripheral enhancing components, which was managed with wide local excision. Rai Bansal et al^5^ have reported a case of primary malignant nerve sheath tumour arising from the retroperitoneum and displacing the surrounding structures, and showing local invasion of the adjacent large bowel. CT imaging in their case showed a large, predominantly cystic mass lesion with peripheral heterogeneously enhancing solid areas and small foci of calcification. Similarly, CT imaging in our case showed an invasive oval mass lesion centred in the omentum with intensely enhancing solid areas and peripheral non-enhancing cystic areas with dilated omental vein draining it (omental vascular pedicle sign). In rare cases, secondary metastatic MPNSTs of the omentum can present as omental caking.^6^

MPNSTs are often seen (up to 50%) in association with neurofibromatosis Type 1 (NF1).^7^ These tumours have been documented to be locally aggressive and recurrent. The recurrence rate after treatment has been noted to vary from 25% to 50%.^7,8^ There are a few case reports of malignant nerve sheath tumour of the peritoneum involving the anterior abdominal wall locally.^7,8^ Of these, in the report by Khorgami et al,^7^ a patient, with a known case of NF1, had local recurrence. There is no standard protocol for management of this condition. Tumour size, total excision, lack of S100 expressivity, absence of local invasion and neurofibromatosis have been seen to portend a better prognosis.^4,7^

Radical surgical resection with post-operative radiotherapy despite having negative surgical margins is currently the recommended form of treatment for MPNST.^5^ MPNST has a high rate of recurrence, as was noted in our case, and adequate initial treatment and close follow up are required to improve survival.^5^

## Conclusions

MPNST of the greater omentum is an uncommon tumour that can show local invasion and has a high risk of recurrence and distant metastases. The knowledge of this uncommon entity is important for radiologists as well as clinicians for its early diagnosis and adequate management. The omental vascular pedicle sign is an important sign that can be demonstrated on a CT scan to help establish the omental origin of intra-abdominal masses, which is important in narrowing down the radiological differential diagnosis and preoperative planning of potentially resectable tumours.

## Learning points

1. Omental vascular pedicle sign helps in localizing an omental mass lesion.
2. Differential diagnoses of primary and secondary neoplasms  of omentum and other generalised diseases affecting the omentum are listed.
3. Peripheral nerve sheath tumour of the omentum should be included in the differential diagnosis of a suspected solitary omental mass.
4. Malignant nerve sheath tumour of omentum has high rate of recurrence; therefore, diagnosis is important for resectable masses. Adequate management and follow up  is advised.**

## Consent

Informed consent has been obtained and is held on record.
